# Tannin profile, antioxidant properties, and antimicrobial activity of extracts from two Mediterranean species of parasitic plant *Cytinus*

**DOI:** 10.1186/s12906-019-2487-7

**Published:** 2019-04-05

**Authors:** Giuseppantonio Maisetta, Giovanna Batoni, Pierluigi Caboni, Semih Esin, Andrea C. Rinaldi, Paolo Zucca

**Affiliations:** 1 Department of Translational Research and New Technologies in Medicine and Surgery, University of Pisa, Pisa, Italy; 2 Department of Life and Environmental Sciences, University of Cagliari, 09124 Cagliari, Italy; 3 Department of Biomedical Sciences, University of Cagliari, Cittadella Universitaria, 09042 Monserrato (CA), Italy

**Keywords:** *Cytinus*, Antimicrobial, Antioxidant, Tyrosinase inhibitors, Anti-biofilm, Gallotannins

## Abstract

**Background:**

*Cytinus* is small genus of endophytic parasitic plants distributed in South Africa, Madagascar, and in the Mediterranean region. In the latter area, two species occur, *Cytinus hypocistis* and *C. ruber*, distinguished by both morphological characters and ecological traits. We characterized the ethanolic and aqueous extracts obtained from the inflorescences of *C. hypocistis* and *C. ruber* collected in Sardinia, Italy, and explored their tannin content, antioxidant properties and antimicrobial activities.

**Methods:**

Total phenolic contents were determined by Folin-Ciocalteu spectrophotometric method. Tannin content was determined by HPLC. Antioxidant activity of the extracts was tested with both electron transfer-based (FRAP, TEAC, DPPH) and spectrophotometric HAT methods (ORAC-PYR). The antimicrobial activities of extracts/compounds were evaluated using the broth microdilution method. The bactericidal activity was evaluated using the time-kill method. Biofilm formation was evaluated by crystal violet (CV) staining assay.

**Results:**

Characterization of the tannin profile of C*. hypocistis* and *C. ruber* revealed a significant amount of gallotannins, in particular 1-*O*-galloyl-β-D-glucose. In addition, pentagalloyl-*O*-β-D-glucose was present in all extracts, reaching the concentration of 0.117 g/kg in the ethanolic extract of *C. hypocistis*. *C. hypocistis* extracts displayed a strongest antioxidant activity than *C. ruber* extracts. Three Gram-positive bacterial species tested (*Staphylococcus aureus*, *Staphylococcus epidermidis*, *Enterococcus faecium*) resulted sensitive to both *Cytinus* extracts, with MICs ranging from 125 to 500 μg/ml for aqueous extracts and from 31.25 to 250 μg/ml for ethanolic extracts; on the contrary, Gram-negative strains (*Pseudomonas aeruginosa* and *Klebsiella pneumoniae*) were not affected by *Cytinus* extracts. Intriguingly, we observed the suppressive activity of ethanolic extracts of *C. hypocistis* and *C. ruber* on biofilm formation of *S. epidermidis*. Experiments performed with synthetic compounds indicated that pentagalloyl-*O*-β-D-glucose is likely to be one of the active antimicrobial components of *Cytinus* extracts.

**Conclusions:**

These findings show that *Cytinus* extracts have antimicrobial and antioxidant activities, suggesting a possible application of *Cytinus* as sources of natural antimicrobials and antioxidants.

## Background

Secondary metabolites of plants are well known to exert health-promoting effects in humans. Phenolics, in particular, are a vast array of plant-derived substances with diversified biological activities, from antioxidant and anticancer properties up to the ability to inhibit and kill selected pathogenic bacteria [1–3].

**Fig. 1:**
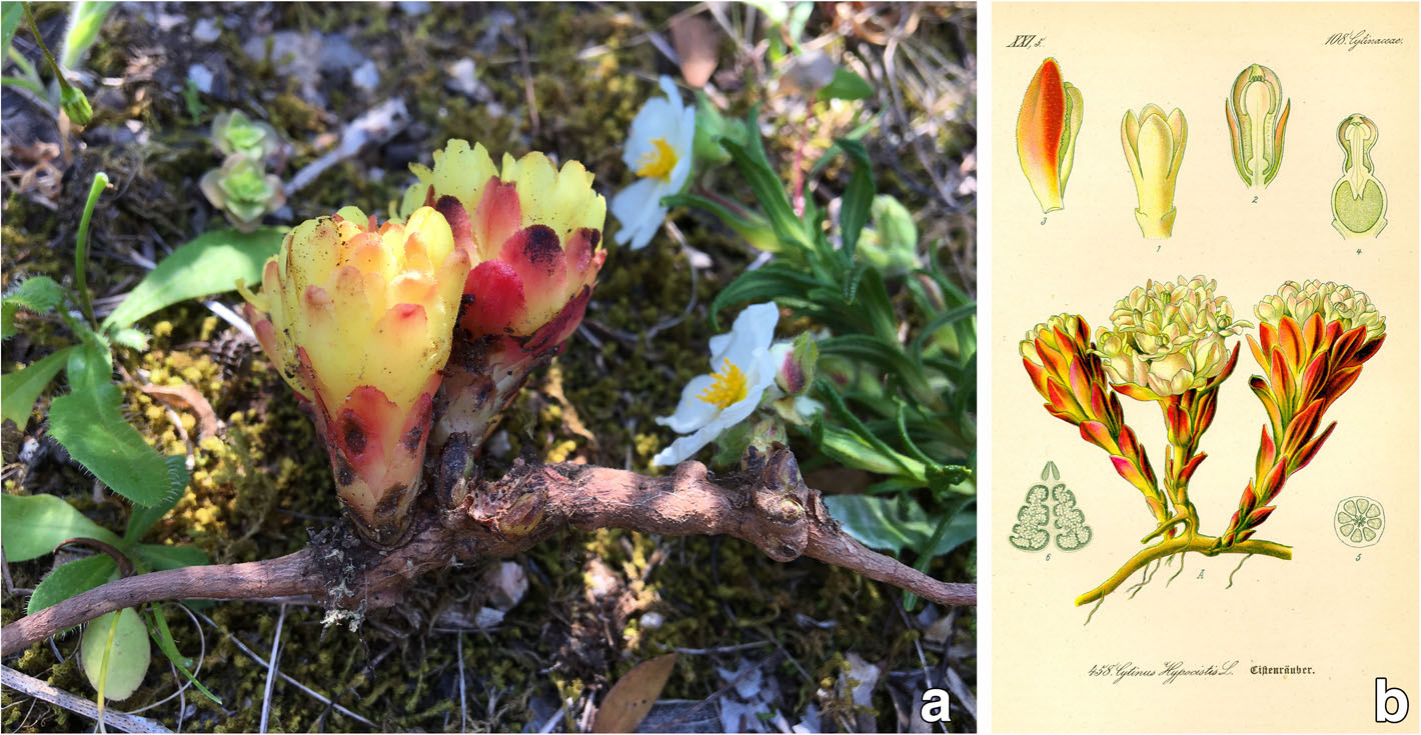
**a** *Cytinus hypocistis* inflorescences, emerging directly from a *Cistus monspeliensis*’ root, Sardinia; (**b**) A classic portrait of *C. hypocistis* (from Reference [52]; in the public domain: https://en.wikipedia.org/wiki/Cytinus#/media/File:Illustration_Cytinus_hypocistis0.jpg)

*Cytinus* (*Cytinaceae*) is a small genus of holoparasitic, nonchlorophyllic plants, with eight recognized species [4]. It grows endophytically, within the tissues of the host plant: flowers are the only visible part, when they emerge from host tissues during the reproductive period (Fig. 1). The genus has a disjunct distribution, with two centers of diversity: one around the Mediterranean and the other in southern Africa and Madagascar [5, 6]. In the Mediterranean area, *Cytinus* parasitize the roots of two genera of shrub plants, *Cistus* and *Halimium*, both belonging to the family *Cistaceae* [7].

*Cytinus* has a place in European popular medicine [8], being used traditionally in the treatment of dysentery, for its a stringentand haemostatic qualities, and for soothing the inflammations of the throat and of eyes (see [9] and references therein, [10]). In Sardinia, ethnobotanical surveys conducted in the south-central part of the island ascertained the *Cytinus* juice was used as an astringent, tonic, and haemostatic substance [11]. “The plant was known for its astringent and tonic properties: the blackish juice, squeezed and condensed, was used to make the concoctions. The astringent property was exploited in places such as Lodè, Lula and Siniscola as anti-hemorrhage, and in Sadali, Seui and Seulo as haemostatic. At Perdasdefogu, the scalp pulp was applied daily on corns and calluses as a scar-healing agent, and on the skin and inflamed mucous membranes as an astringent and anti-inflammatory remedy,” reports Atzei [12] on the ethnobotanical uses of *Cytinus* in Sardinia.

**Fig. 2:**
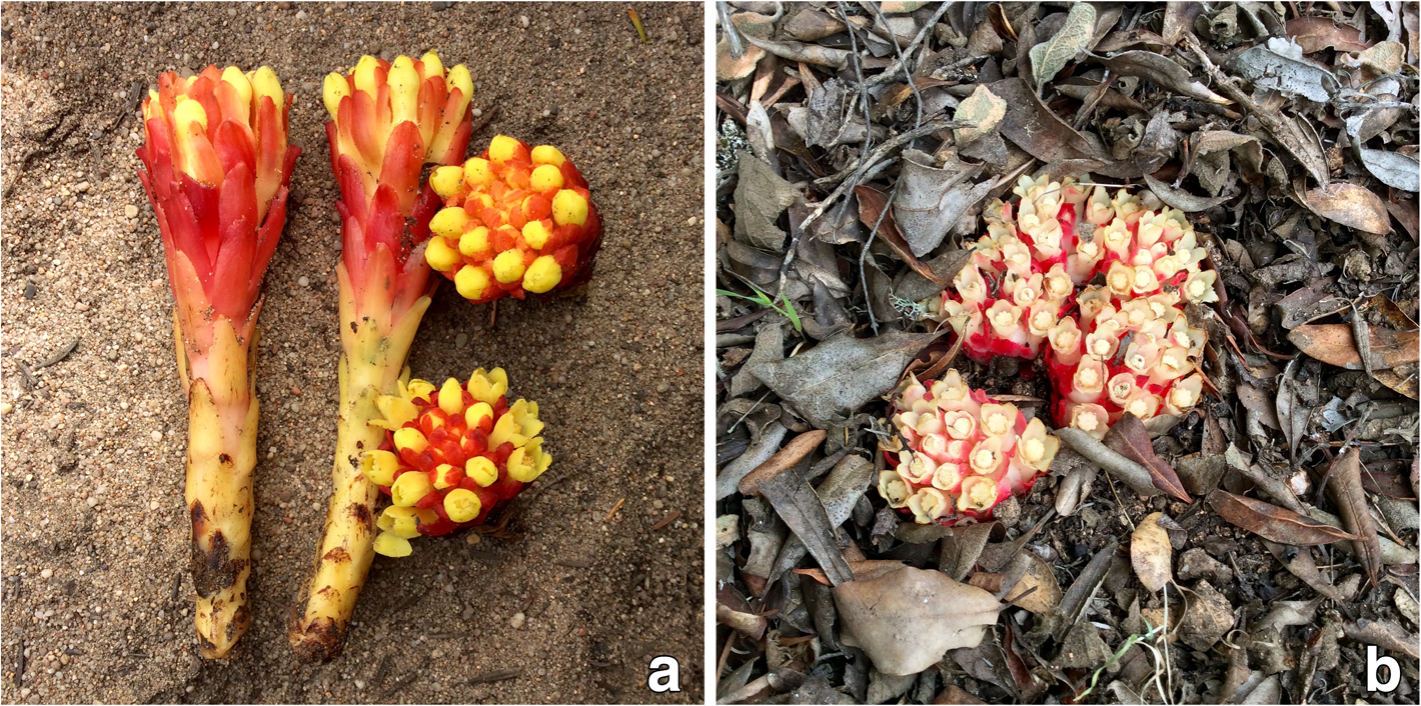
**a** *Cytinus hypocistis* inflorescences, Sardinia; (**b**) *Cytinus ruber* inflorescences, Sardinia

As for many plants used in traditional medicine, the real biological activities of *Cytinus* are largely unknown or not rigorously measured, and active substances not identified. Previous reports [13, 14], have described antimalarial and antitumoral activities of extracts of *C. hypocistis* (Hypoquisitis, Hipocistide, Melera, Chupamiele, Cytinet, Cytinelle, Ipocisto, Rockrose parasite, Cistenräuber), and preliminarily assessed their antimicrobial, antioxidant, and anti-tyrosinase properties [9]. To extend our knowledge of the chemical composition of *Cytinus*, and to explore alternative potential medicinal uses of this plant, we here describe the tannin profile and antioxidant properties of extracts of *C. hypocistis* (L.) L. and *C. ruber* (Fourr.) Fritsch (synonym *C. clusii*, *C. hypocistis* subsp. *clusii*) collected in Sardinia (Figs. 1 and 2), Italy, and describe in detail their antimicrobial and anti-biofilm activities.

## Methods

### Chemicals

All reagents were of the best commercial grade available and used without further purification. Tannin standards 1-*O*-galloyl-β-D-glucose and penta-*O*-galloyl-β-D-glucose were purchased from Sigma-Aldrich (Milan, Italy). Ethanol, cyclohexane and dimethyl sulfoxide were analytical grade solvents obtained from Sigma-Aldrich, Fluka (Milan, Italy).

### Plant materials

*Cytinus hypocistis* and *C. ruber* were collected in a mountainous area (Sette Fratelli) 30 km east of Cagliari, Sardinia, Italy, during April 2016 and April 2017. Plants were identified following field guides and identity confirmed by specialized personnel at the Herbarium of the University of Cagliari, Cagliari, Italy (CAG). Reference material for *C. hypocistis* (ACR-Cythyp/2016/1,ACR-Cythyp/2017/2) and *C. ruber* (ACR-Cytrub/2016/3,ACR-Cytrub/2017/4) is deposited in the collection of the Department of Biomedical Sciences, University of Cagliari. After harvesting, the samples were cleaned, and frozen within 1 h and then freeze-dried. The samples were grinded afterwards.

### Preparation of the extracts

Samples were resuspended in cyclohexane (1 g in 50 mL solvent). After 30 min of magnetic stirring at 25 °C, the suspensions were centrifuged at 5000 *g* for 10 min. The extraction was repeated 4 times, combining the extracts. Rotary evaporation was used to remove cyclohexane, and to dry the remaining sample. The whole extraction process was then repeated using ethanol, and lastly water as solvents, to obtain three extracts at increasing polarity [9].

### Phenolics determination

Folin-Ciocalteu reagent was used for the spectrophotometric quantification of total phenolics, as already described [15], using an UltroSpec 2100pro (Amersham Bioscience, Milan, Italy). Briefly, 2.5 ml Na_2_CO_3_ 2% *w*/*v* and 1 ml of each sample were incubated for 1 min at 25 °C. Then, 45 min incubation in the dark at 25 °C with 0.25 ml 1 N Folin-Ciocalteu reagent followed. Absorbance at 760 nm was then recorded. The results were expressed as gallic acid equivalents (mmol GAE). Total flavonoids were quantified using the reaction between sodium nitrite and aluminium chloride [16, 17]. Briefly, 0.25 ml of sample, 1.25 ml H_2_O and 0.075 ml NaNO_2_ (5% *w/v*) were incubated for 5 min at 25 °C, then 0.15 ml AlCl_3_ (10% *w/v*) was added. After 6 min, the reaction mixture was alkalinized with 0.5 mL of 1 M NaOH and 0.275 ml H_2_O. Finally, the absorbance was recorded at 510 nm. Results were expressed as Catechin Equivalent (mmol CE). Differential pH absorbance method was used to quantify total anthocyanins [18]. Briefly, absorbance at pH 1.0 and 4.5 and 510 nm and 700 nm were measured. HCl/KCl 0.2 M and sodium acetate 1 M buffers were used to achieve thoss pH values. Molar extinction coefficient of cyanidin 3-*O*-glucoside (29,300 M^− 1^ cm^− 1^) was used to calculate total anthocyanin.

1 $$ \left[ total\ anthocyanin\right]=\frac{\left[{\left({A}_{510}-{A}_{700}\right)}_{pH1.0}-{\left({A}_{510}-{A}_{700}\right)}_{pH4.5}\right]}{\mathrm{29,300}\ {M}^{-1}{cm}^{-1}\bullet l} $$

### Tannin profile

The *Cytinus* extracts dissolved in a (50:50 *v/v*) solution of methanol and water at the concentration of 1000 mg/l were analyzed by reverse phase HPLC on an Agilent 1200 series HPLC system fitted with an Agilent, Zorbax C18 (4.6 × 100 mm, 3 μm). The HPLC conditions were as follows: flow rate, 0.4 ml/min; solvent A, 0.1% formic acid in water; solvent B, methanol; gradient, solvent B 20–100% over 10 min and kept at 100% for 10 min. The extract was injected (4 μL) in the HPLC system and analyzed by ESI-QTOF-MS in the negative mode using an Agilent 6520 time-of-flight mass spectrometer. Mass spectral data were acquired in the range m/z 100–3000, with an acquisition rate of 1.35 spectra/s, averaging 10,000 transients. The source parameters were adjusted as follows: drying gas temperature 250 °C, drying gas flow rate 5 L/min, nebulizer pressure 45 psi, and fragmentor voltage 150 V. Data acquisition and processing were done using Agilent Mass Hunter Workstation Acquisition v. B.02.00 software.

### Determination of antioxidant capacity

Three electron-transfer methods were used for the determination of antioxidant power of the samples: 1,1-Diphenyl-2-picrylhydrazyl radical (DPPH) scavenging assay, Ferric Reducing Antioxidant Power (FRAP), and Trolox Equivalent Antioxidant Capacity (TEAC) assay.

DPPH assay involved 30 min incubation of 0.7 ml of ethanolic 25 mg/l DPPH solution and 0.3 mL of sample. Absorbance at 515 nm was the measured and converted in DPPH decoloration (%_DEC_): %_DEC_ = 100 x [(Abs_control_- Abs_sample_)/Abs_control_]. Trolox was used for the calibration curve (linearity range 5–50 μM) [17]. FRAP method was performed by adding 2.5 ml of 10 mM 2,4,6-tripyridyl-*s*-triazine (TPTZ) in 40 mM HCl, 25 mL of 0.1 M sodium acetate buffer (pH 3.6) and 2.5 ml of 20 mM FeCl_3_. After an incubation at 37 °C, 0.03 ml of sample was then treated for 6 min at 25 °C with 0.2 ml of this solution and 0.77 ml H_2_O. After 10 min centrifugation at 8000 g for 10 min, absorbance at 593 nm was read [19]. Both Trolox and Fe (II) were used for the calibration curves. TEAC assay involved 2,2′-azinobis (3-ethylbenzothiazoline 6-sulphonate) (ABTS) cationic radical. This was synthesized starting from 7 mmol aqueous ABTS and 2.45 mmol aqueous K_2_S_2_O_8_. After 16 h reaction at 25 °C, the radical was produced. Before use, the green radical was diluted using sodium phosphate buffer 75 mM (pH 7.4) to absorbance 0.70 ± 0.01 (at 734 nm). Then, 0.01 ml of samples were treated with 1 mL of this ABTS radical. Absorbance at 734 nm was the recorded after 6 min reaction at 25 °C [17], and converted in percentage of ABTS decoloration (%DEC): %DEC = 100 x [(Abs_control_- Abs_sample_)/Abs_control_]. Trolox was used for the calibration curve.

One hydrogen atom transfer method was also included in the antioxidant screening: Oxygen Radical Absorbance Capacity-Pyrogallol red (ORAC-PYR) assay. Briefly, 6.6 mM pyrogallol red (0.75 ml) was incubatedat 25 °C for 10 min with 0.125 mL of the sample. Then 0.125 ml of 0.153 mM 2,2′-azobis (2-amidinopropane) dihydrochloride (APH) was added, recording the decrease in absorbance at 540 nm for 35 min at 25 °C. The area under the kinetic curves was determined using Origin Pro software (Origin Lab Corporation, Northampton, Massachusetts, USA): using the area of the blank (AUC_blank_): AUC_net_ = AUC_sample_ - AUC_blank_ [16]. Trolox was used for the calibration curve.

### Tyrosinase inhibition

Tyrosinase inhibition was determined using purified enzyme from *Agaricus bisporus* [20]. Laccase activity was not presentin the final preparation (< 0.001 E.U./ml) [21], using syringaldazine as the substrate [22], confirming the absence of contaminant and disturbing activities [23]. 4-*tert*-Butylcatechol (TBC) was chosen as the substrate, using 4-amino-*N*,*N*-diethylaniline (ADA) to enhance sensibility [24], in fact these yield a blue adduct upon tyrosinase oxidation, with a maximum wavelength quite far from the extracts. The reaction was performed in the presence of 2 E.U. of enzyme, 50 mM sodium phosphate buffer pH 7.0, 5 mM TBC, 0.75 mM ADA in a final volume of 1 mL. The absorbance at 625 nm (ε_625_ = 11,120 M^− 1^ cm^− 1^) was followed. One tyrosinase E.U. was the amount of enzyme capable of producing 1 μmol of product per minute at pH 7 and 25 °C. The amount of inhibition by the test samples was expressed IC_50_.

### Bacterial strains and culture conditions

The following bacterial species/strains were used for the study: *Staphylococcus aureus* (ATCC 33591), *Staphylococcus epidermidis* (ATCC 35984), *Pseudomonas aeruginosa* (ATCC 27853), *Klebsiella pneumoniae* (ATCC BAA-1706) and the clinical isolate *Enterococcus faecium* VanR 1. For liquid culture, bacteria were grown in Luria Bertani broth (LB), in Mueller Hinton Broth (MHB) or in Tryptone Soy Broth (TSB) (Oxoid, Basingstoke, UK) at 37 °C with shaking depending on the type of experiment. Enumeration of colony-forming units (CFU) was performed by serially diluting bacterial suspensions and plating them on Tryptone Soy Agar (TSA) (Oxoid). After an incubation of 24 h at 37 °C, CFU were counted.

### Broth microdilution assay

Determination of the minimum inhibitory concentration (MIC) was performed according to standard procedures [25]. Briefly, bacterial strains were grown in Muller–Hinton broth (MHB) until exponential growth phase and diluted with fresh MHB to reach a density of 5×10^6^ CFU/ml. Bacterial suspensions (10 μl) were added to 96-well microtiter plates containing 90 μl of *Cytinus* extracts or synthetic galloyl compounds at different concentrations and incubated for 24 h at 37 °C. DMSO at 2.5% was used as solvent in the control. The MIC was defined as the lowest concentration of the tested compounds that prevented the visible growth of bacteria.

### Bactericidal and time killing assay

The bactericidal activity of *Cytinus* extracts and of synthetic penta-*O*-galloyl-β-D-glucose was evaluated against *S. aureus* ATCC 33591 and *S. epidermidis* ATCC 35984 in sodium-phosphate buffer (SPB, 10 mM, pH 7.4) supplemented with 2% LB (SPLB). Overnight cultures were diluted 1:100 in LB and then were incubated at 37 °C to achieve exponential growth phase. The suspensions were then diluted in SPLB to reach a density of 1 × 10^7^ CFU/ml. To identify the bactericidal concentrations of the extracts in SPLB, in preliminary experiments a volume of 10 μl of the bacterial suspensions was added to 90 μl of SPLB containing different concentrations of *C. hypocistis* or *C. ruber* extract. Bacteria suspended in SPLB alone were used as cell viability control. For the time killing assays, test samples were incubated with the identified concentrations of each extract at 37 °C with shaking for 3 h and 24 h, respectively. Following incubation, samples were 10-fold diluted in LB and plated on TSA to determine the number of CFU. Bactericidal activity was defined as a reduction of at least 3 Log_10_ in the number of viable bacteria as compared to the inoculum.

### Biofilm inhibition assay

The *S. epidermidis* ATCC 35984 strain was cultured overnight and then diluted 1:1000 in TSB. Bacterial suspension was dispensed into wells of a flat-bottom polystyrene 96-well microplates (Corning Costar, Lowell, USA), in the presence of each extract or tannin compound used at the concentration of 1/2 MIC. Bacteria incubated in the absence of the compounds represented negative controls. After a static incubation of the microplates at 37 °C for 24 h, biofilm biomass was quantified by crystal violet (CV) staining assay. Briefly, biofilms were washed three times with phosphate-buffer saline (PBS), dried for 1 h at 60 °C and incubated for 15 min with 1% (*w*/*v*) CV (bioMérieux, Florence, Italy). The plates were extensively washed with PBS to remove the unbound CV. Plates were then dried at 37 °C for 30 min. Biofilm-associated CV was extracted with 33% acid acetic (Sigma Aldrich) and measured by evaluating the optical density at 570 nm (OD_570_) in a microplate reader (Model 550, Bio-Rad Laboratories Srl, Italy).

### Statistical analysis

Grafit 7 (Erithacus Software, London UK), and R 2.5.1 software (R Foundation for Statistical Computing, Vienna), were used to statistical analysis. All analyses were performed in triplicate, if not differently stated. Microbiological data are reported as mean ± standard error of the mean of at least three independent experiments. The statistical significance of the data was evaluated by one-way ANOVA followed by Tukey-Kramer post hoc test. A *p* value < 0.05 was considered significant.

## Results

### Phenolics content and antioxidant activity of *Cytinus* extracts

To ascertain the chemical composition of *C. hypocistis *and *C. ruber* we used three sequential extraction steps with increasingly polar solvents to fractionate the freeze dried plants: cyclohexane, ethanol, and water. While in both cases the hydrophobic portion of the plant was minimal (data not shown), ethanol and water allowed significant recovery (Table 1). The polyphenolic component of the extracts was quantified, as well as total flavonoids and anthocyanins. For both *Cytinus* species the ethanolic extract was the richest fraction, with significantly more phenolics than the water analogue. Flavonoids accounted for only a small part of total phenolics, whereas no anthocyanins were detected (Table 1). Antioxidant was then determined using three different spectrophotometric electron transfer-based methods (TEAC-ABTS, FRAP, and DPPH-scavenging) and a HAT method (ORAC-PYR). In all cases, *C. hypocistis* extracts displayed a strongest antioxidant activity than *C. ruber* extracts, both when results were expressed as Trolox Equivalents (mM TE/g) and as IC_50_ (Table 1).

**Table 1:** Total antioxidant capacity of *Cytinus hypocistis* and *Cytinus ruber* extracts

Assay	*Cytinus hypocistis*		*Cytinus ruber*	
	Ethanol extract	Water extract	Ethanol extract	Water extract
Amount (g/100 g of dried plant material)	21%	14%	13%	35%
ORAC-PYR (mmol TE/g)	9.1 ± 1.4	7.8 ± 1.1	7.5 ± 1.2	5.1 ± 0.8
DPPH (mmol TE/g)	6.2 ± 0.6	4.5 ± 0.1	4.0 ± 0.5	2.6 ± 0.2
DPPH (IC_50_ μg/mL)	6.8 ± 1.2	8.2 ± 0.4	11.4 ± 0.6	15.8 ± 0.7
TEAC (mmol TE/g)	11.2 ± 0.8	7.8 ± 0.3	6.5 ± 0.7	5.6 ± 0.3
TEAC (IC_50_ μg/mL)	83 ± 9	121 ± 13	140 ± 15	168 ± 21
FRAP (mmol TE/g)	8.4 ± 0.4	5.2 ± 0.6	5.9± 0.2	3.8 ± 0.1
FRAP (mmol Fe^II^/g)	9.3 ± 0.9	6.5 ± 0.1	5.2 ± 0.3	4.7 ± 0.1
Total phenolics (mmol GAE/g)	12.2 ± 0.1	8.88 ± 0.07	9.57 ± 0.09	6.27 ± 0.02
Total flavonoids (mmol RE/g)	0.48 ± 0.01	0.38 ± 0.03	0.51 ± 0.04	0.27 ± 0.01
Total anthocyanins (mg cyanidin 3-*O*-glucoside/g)	n.d.	n.d.	n.d.	n.d.

Data are expressed per gram of dry extract; *n.d.* not detectable

Tyrosinase (or polyphenol oxidase PPO) is a well-known enzyme involved in melanogenesis and food browning. The development of new inhibitors of this enzymatic activity is important in the perspective of application in food formulations as anti-browning agents. To avoid any interference with the tyrosinase inhibition tests, both mono- and di-phenolase activity was ruled out in the *Cytinus* extracts tested using an *ad hoc* assay (see Methods). All the extracts were able to inhibit tyrosinase activity, albeit to different extents. Particularly, both the ethanolic and water extracts of *C. hypocistis* were more effective than the correspondent extracts of *C. ruber* (Table 2). In all cases, ethanolic extracts displayed the strongest anti-tyrosinase activity. The ethanolic extract of *C. hypocistis* had the lowest IC_50_ (9.8 μg), a value that almost doubled in the case of *C. ruber* (IC_50_ 16 μg) (Table 2).

**Table 2:** Anti-tyrosinase activity of *Cytinus hypocistis* and *Cytinus ruber* extracts

Species	Ethanol extract	Water extract
*Cytinus hypocystis*	9.8 ± 0.7	20 ± 2
*Cytinus ruber*	16 ± 2	33 ± 6

Data expressed as IC_50_ (μg)

### *Cytinus* tannin profile

Analysis of the extracts of *C. hypocistis* and *C. ruber* through HPLC and MS revealed that they contained a significant amount of gall tannins (Table 3). β-Glucogallin (1-O-galloyl-β-D-glucose) was particularly abundant, reaching almost 20 g/kg in the water extract of *C. hypocistis*. In general, *C. hypocistis* extracts contained an higher amount of tannins with respect to *C. ruber*. We confirmed the presence of pentagalloyl-O-β-D-glucose in all extracts, which reached the concentration of 0.117 g/kg in the ethanolic extract of *C. hypocistis* (Table 3). The only possible comparison can be made with a previous study of the chemical composition and cytotoxic properties of extracts of *Cytinus* collected in Grece [13]. In this work, samples of *Cytinus ruber* were analyzed and hydrolysable tannins (including 1,2,3,6-tetragalloyl-O-β-D-glucose and 1,2,3,4,6-pentagalloyl-O-β-D-glucose) were identified as the main component [24]. In a further study, isoterchebin, another hydrolysable tannin of the ellagitannin class, was determined to be at the origin of the yellow pigment of *C. hypocistis* [26].

**Table 3:** Levels of tannins in *Cytinushypocistis* and *Cytinusruber* extracts (g/kg)

Tannin	*Cytinus hypocistis* (Ethanol)	*Cytinus hypocistis* (Water)	*Cytinus ruber* (Ethanol)	*Cytinus ruber* (Water)
1-O-galloyl-β-D-glucose	9.991	19.292	2.608	1.994
di-O-galloyl-β-D-glucose	0.281	0.846	0.054	0.119
tri-O-galloyl-β-D-glucose	0.255	0.316	0.145	0.057
tetra-*O*-galloyl-β-D-glucose	0.670	0.078	0.637	0.060
penta-O-galloyl-β-D-glucose	0.117	0.073	0.043	0.195
Tellimagradin	0.063	0.039	–	–

CV < 5%

### Minimum inhibitory concentrations of *Cytinus* extracts

The MIC values of aqueous and ethanolic extracts of both *C. hypocistis* and *C. ruber* against relevant pathogenic bacterial species were determined in MHB. Synthetic 1-O-galloyl-β-D-glucose and penta-O-galloyl-β-D-glucose, whose presence was demonstrated in both *Cytinus* extracts, were also tested for their antimicrobial properties. All three Gram-positive bacterial species tested resulted sensitive to both *Cytinus* extracts (Table 4). In particular, aqueous extracts exhibited MICs ranging from 125 to 500 μg/ml whereas MICs of ethanolic extracts ranged from 31.25 to 250 μg/ml (Table 4). The compound penta-O-galloyl-β-D-glucose resulted active towards the same Gram-positive species with MIC values ranging from 31.25 to 62.5 μg/ml, whereas 1-O-galloyl-β-D-glucose was inactive up to the concentration of 500 μg/ml. In contrast to the Gram-positive bacteria, any of the extracts tested up to 500 μg/ml was able to inhibit the growth of the two Gram-negative species (Table 4).

**Table 4:** Antimicrobial activities of water and ethanolic extracts of *Cytinus* against Gram-positive and Gram-negative bacteria

	*Cytinus hypocistis*		*Cytinus ruber*		1-O-galloyl-β-D-glucose	penta-O-galloyl-β-D-glucose
Bacterial strains	Water extract	Ethanolic extract	Water extract	Ethanolic extract		
Gram-positive						
*S. aureus* ATCC 33591	500	125	250	125	> 500	62.5
*S. epidermidis* ATCC 35984	500	250	250	250	> 500	62.5
*E. faecium* VanR 1	125	31.25	125	31.25	> 500	31.25
Gram-negative						
*P. aeruginosa* ATCC 29534	> 500	> 500	> 500	> 500	> 500	> 500
*K. pneumoniae* (ATCC BAA-1706)	> 500	> 500	> 500	> 500	> 500	> 500

Data reported represent minimal inhibitory concentrations (MIC) values expressed in μg/ml

### Bactericidal activity of *Cytinus* extracts

**Fig. 3:**
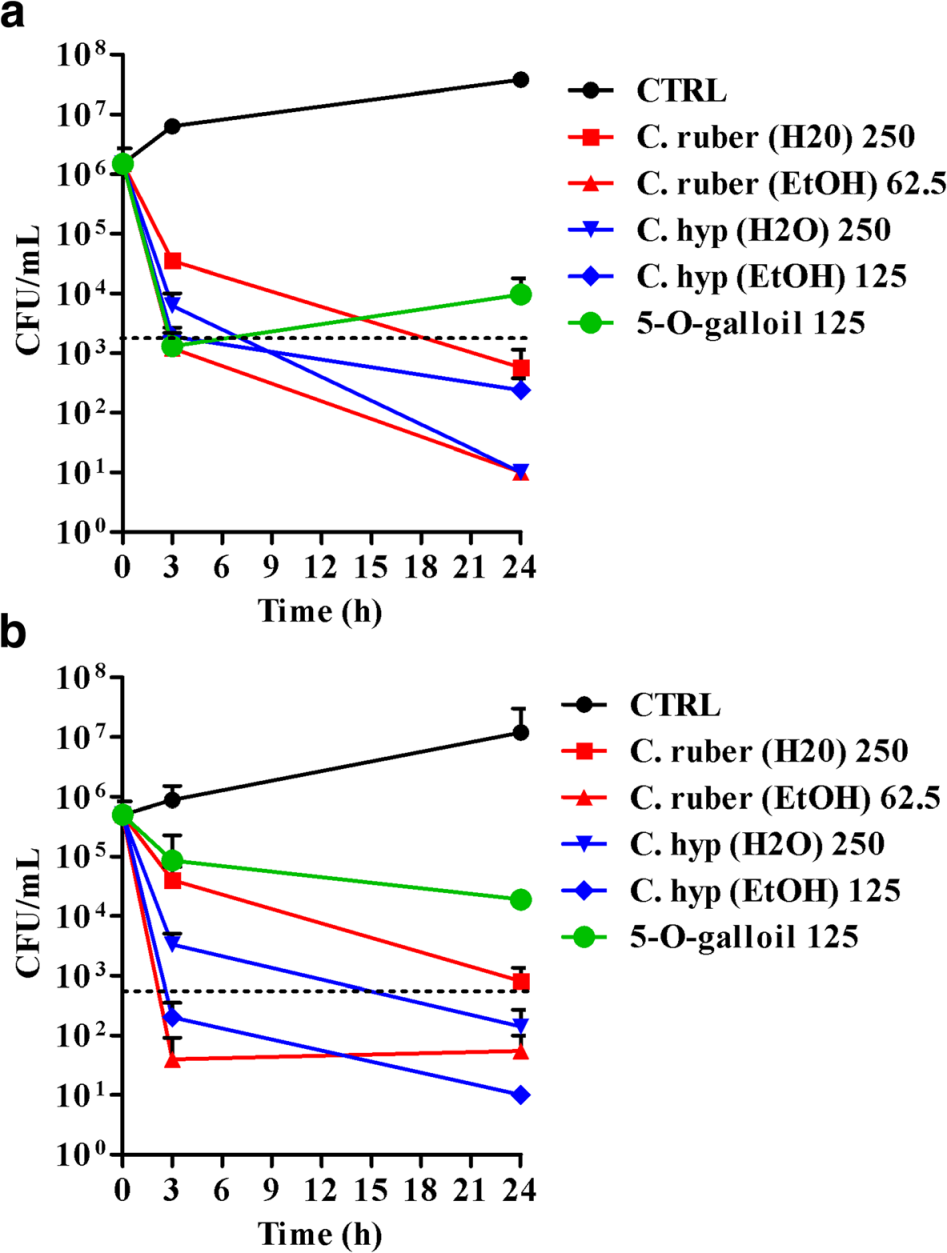
Killing kinetics of *Cytinus hypocystis* and *Cytinus ruber* extracts (at concentrations ranging from 62.5 to 250 μg/ml) and penta-O-galloyl-β-D-glucose (abbreviated as 5-O-galloyl in the graphs, 125 μg/ml) against *S. aureus* ATCC 33591 (**a**) and *S. epidermidis* ATCC 35984 (**b**). Bacteria were incubated in sodium-phosphate buffer 10 mM, pH 7.4 supplemented with 2% LB in the presence of the antimicrobial compounds for 3 and 24 h. Control (CTRL) represents untreated bacteria. Dashed line represent 3 Logs reduction in CFU count as compared to inoculums. The numbers in the figure legend are the concentrations used expressed in μg/ml. Data are expressed as mean ± standard error of at least three independent experiments

The bactericidal activity of the aqueous and ethanolic extracts of *C. hypocistis* and *C. ruber* as well as of synthetic penta-O-galloyl-β-D-glucose was evaluated against *S. aureus* ATCC 33591 and *S. epidermidis* ATCC 35984 in SPLB. As shown in Fig. 3, after 24 h of incubation all the extracts tested resulted bactericidal against both bacterial species causing approximately 3 Logs reduction in the number of viable cells at concentrations ranging from 62.5 to 250 μg/ml. At such concentrations, the killing effect was evident also at 3 h of incubation with reductions in the number of CFUs ranging from 1 and 3 Logs depending on the extract and the bacterial species tested. Synthetic penta-O-galloyl-β-D-glucose was bactericidal against *S. aureus* at 3 h incubation, while only an 1.5 Log reduction in the number of CFU at 24 h was recorded against *S. epidermidis*.

### Anti-biofilm activity of *Cytinus* extracts against *S. epidermidis*

**Fig. 4:**
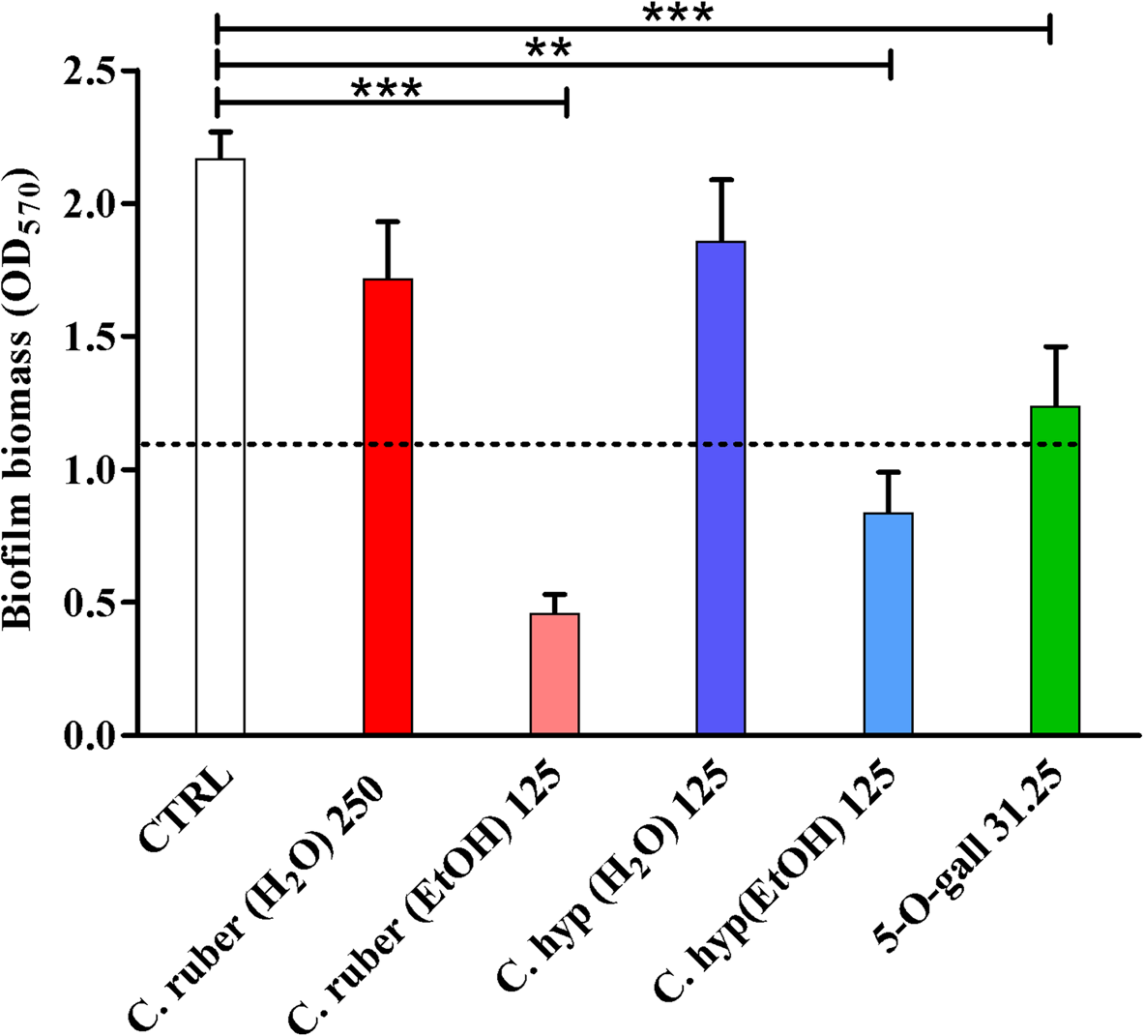
Inhibitory effect of *Cytinus hypocystis* and *Cytinus ruber* extracts (at concentrations ranging from 125 to 250 μg/ml) and penta-O-galloyl-β-D-glucose (abbreviated as 5-O-gall in the graph; 31.25 μg/ml) on biofilm formation of *S. epidermidis* ATCC 35984. The inhibitory effect was assessed by measuring the total biofilm biomass by crystal violet staining after 24 h of incubation with the extracts. Control (CTRL) represents untreated bacteria. Concentrations are expressed in μg/mL. Dashed lines represent 50% reduction in biofilm biomass as compared to untreated controls. Data are reported as mean ± standard error of at least three independent experiments. ∗∗*p* < 0.01; ∗∗∗*p* < 0.001 (one-way ANOVA followed by Tukey-Kramer post hoc test)

Next, we investigated the ability of *C. hypocistis* and *C. ruber* extracts as well as of the two synthetic phytochemicals 1-O-galloyl-β-D-glucose and penta-O-galloyl-β-D-glucose to inhibit the formation of biofilms of *S. epidermidis*, one of the major nosocomial pathogens, often involved in medical device-associated infections [27]. Ability of the bacterium to colonize and form biofilms on a variety of biotic and abiotic surfaces is regarded as one of the main virulence factor of such bacterial species urging the identification of new antimicrobials with anti-biofilm potential [28]. The biofilm inhibitory effect was assessed after 24 h incubation with *Cytinus* extracts by CV staining, a technique that allows the evaluation of the total biofilm biomass (extracellular matrix and biofilm-associated cells). As shown in Fig. 4, ethanolic extract of both *C. hypocistis* and *C. ruber*, tested at sub-inhibitory concentrations (1/2 MIC), caused an inhibitory effect in biofilm formation of 80 and 60%, respectively as compared to the control biofilms (cells incubated in medium only). In contrast, water extracts of both *Cytinus* species did not display a statistically significant reduction of biofilm biomass (Fig. 4) at the tested concentrations. Penta-O-galloyl-β-D-glucose was able to cause a reduction of biofilm formation of approximately 45%, whereas 1-O-β-D-galloyl-glucose was completely inactive (data not shown).

## Discussion

*Cytinus hypocistis* and *C. ruber* are the only two species of this genus present in the Mediterranean area. These parasitic plants are easily distinguished in the field: *C. hypocistis* has bright yellow flowers while *C. ruber* has flowers with crimson or bright-red scale leaves and bracts, and an ivory-white or pale pink perianth (Figs. 1 and 2). Moreover, the two species occupy different ecological niches, as *C. hypocistis* is parasitic on white-flowered *Cistus* species (like *C. monspeliensis* and *C. salviifolius*), whereas *C. ruber* occurs on pink-flowered *Cistus* (for example *C. creticus* and *C. albidus*) [29–31]. Plants have developed sophisticated defense mechanisms that allow them to survive in their ecosystems, and hence, they represent a rich source of antimicrobial agents and other compounds of pharmaceutical interest [32, 33]. Over the past decade, the efficacies of several plant derived inhibitors have been investigated to determine their antimicrobial potential and ability to reduce the formation of biofilms of staphylococci [34–36] and other pathogenic bacteria. The results of antimicrobial assays of both *Cytinus* extracts are consistent with previous studies investigating the antimicrobial activity of tannin-rich plant extracts or penta-O-galloyl-β-D-glucose that reported a higher susceptibility of Gram-positive bacteria than Gram-negative bacteria to such extracts/compounds [37, 38]. It has been suggested that the antimicrobial activity of gallo tannins maybe related to their action on the membranes of the bacteria and/or their ability to complex metal ions [39, 40]. The poor antimicrobial activity of tannins against Gram-negative bacteria has been previously attributed to the strong repulsive negative charge of lipopolysaccharides [41]. Furthermore, the bactericidal effect displayed by the *Cytinus* extracts may be due to the presence of tannins, for which a killing activity was previously reported [39, 42, 43].

Relevant to the present investigation, gallo tannins have been identified as a significant part of plants’ active components, playing multiple roles. More specifically, penta-O-galloyl-β-D-glucose raised considerable interest because of its valuable functional properties and potential application as an antimicrobial, anti-inflammatory, antidiabetic and antioxidant agent [44–46]. Besides being active against Gram-positive bacterial strains in planktonic form, penta-O-galloyl-β-D-glucose, either in a solution or coated on solid surfaces, was shown to be able to inhibit biofilm formation by *S. aureus* by inhibiting bacterial attachment and formation of polysaccharide intercellular adhesin [45]. In the present study, we demonstrated for the first time the suppressive activity of ethanolic extracts of *C. hypocistis* and *C. ruber* on biofilm formation of *S. epidermidis*. Interestingly, the antibiofilm activity was observed at sub-inhibiting concentrations, suggesting that the inhibitory effect is not due to a direct antibacterial effect, but rather to a more specific anti-biofilm mechanism [47]. Previous studies demonstrated that tannins – that occur abundantly in *Cytinus* extracts – are able to inhibit the formation of *S. aureus* biofilms by repressing the ica operon, which regulates the synthesis of the biofilm extracellular polysaccharides [48]. The ica operon is also present in *S. epidermidis* ATCC 35,984 and has an important role in biofilm formation [49, 50]. Thus, it can be presumed that *Cytinus* extracts may repress biofilm formation of *S. epidermidis* by inhibiting the synthesis of extra cellular polysaccharides. While no major difference was observed in antibacterial and antibiofilm activity between *C. hypocystis* and *C. ruber* extracts, overall the ethanolic *Cytinus* extracts resulted more active than water extracts in anti-bacterial and anti-biofilm activity. Such difference may be due to the higher phenolic and flavonoid content in ethanolic extracts than water extracts. In addition, it has to be taken into account that additive and/or synergistic effects of multiple phytochemicals can be present in plant extracts [51]. In this regard, further studies will be needed to identify the most effective anti-bacterial combinations of phytochemicals contained in *Cytinus* extracts. The control experiments performed during our investigation by using synthetic gall tannins permit to conclude that penta-O-galloyl-β-D-glucose, present in all *Cytinus* extracts, is an active component, while the abundant 1-O-galloyl-β-D-glucose proved deprived of any activity.

## Conclusions

Overall, the results obtained in the present work reveal a high antibacterial and anti-biofilm efficacy of *C. hypocistis* and *C. ruber* extracts against Gram-positive human pathogens, therefore such extracts may be ranked among natural agents with promising therapeutic potential.

## Abbreviations

ABTS: 2,2′-azinobis (3-ethylbenzothiazoline 6-sulphonate)
ADA: 4-amino-N,N-diethylaniline
APH: 2,2′-azobis (2-amidinopropane) dihydrochloride
CFU: Colony forming unit
CV: Crystal violet
DPPH: 1,1-Diphenyl-2-picrylhydrazyl
ESI: Electrospray ionization
FRAP: Ferric Reducing Antioxidant Power
LB: Luria –Bertani broth
MHB: Muller-Hinton broth
MIC: Minimal inhibitory concentration
MS: Mass spectrometry
ORAC-PYR: Oxygen Radical Absorbance Capacity-Pyrogallol red
SPB: Sodium phosphate buffer 10 mM pH 7.4
SPLB: SPB supplemented with 2% LB
TBC: 4-tert-butylcatechol
TEAC: Trolox Equivalent Antioxidant Capacity
TOF: Time of flight
TSA: Tryptone Soy Agar
TSB: Tryptone Soy broth
